# 4-Hydroxyderricin Promotes Apoptosis and Cell Cycle Arrest through Regulating PI3K/AKT/mTOR Pathway in Hepatocellular Cells

**DOI:** 10.3390/foods10092036

**Published:** 2021-08-29

**Authors:** Xiang Gao, Yuhuan Jiang, Qi Xu, Feng Liu, Xuening Pang, Mingji Wang, Qun Li, Zichao Li

**Affiliations:** 1 Institute of Biomedical Engineering, College of Life Sciences, Qingdao University, Qingdao 266071, China; gaoxiang@qdu.edu.cn (X.G.); 2018025210@qdu.edu.cn (Y.J.); xq@qdu.edu.cn (Q.X.); 2018025216@qdu.edu.cn (X.P.); 2 Department of Horticultural Technology, Ningbo City College of Vocational Technology, Ningbo 315199, China; coy24@163.com; 3 Joint Institute of *Angelica keiskei* Health Industry Technology, Qingdao University, Qingdao 266071, China; 13589228558@139.com (M.W.); qunli@qdu.edu.cn (Q.L.); 4 College of Chemistry and Chemical Engineering, Qingdao University, Qingdao 266071, China

**Keywords:** *Angelica keiskei*, chalcone, anti-tumor, mechanism, apoptosis, cell cycle

## Abstract

4-hydroxyderricin (4-HD), as a natural flavonoid compound derived from *Angelica keiskei*, has largely unknown inhibition and mechanisms on liver cancer. Herein, we investigated the inhibitory effects of 4-HD on hepatocellular carcinoma (HCC) cells and clarified the potential mechanisms by exploring apoptosis and cell cycle arrest mediated via the PI3K/AKT/mTOR signaling pathway. Our results show that 4-HD treatment dramatically decreased the survival rate and activities of HepG2 and Huh7 cells. The protein expressions of apoptosis-related genes significantly increased, while those related to the cell cycle were decreased by 4-HD. 4-HD also down-regulated PI3K, p-PI3K, p-AKT, and p-mTOR protein expression. Moreover, PI3K inhibitor (LY294002) enhanced the promoting effect of 4-HD on apoptosis and cell cycle arrest in HCC cells. Consequently, we demonstrate that 4-HD can suppress the proliferation of HCC cells by promoting the PI3K/AKT/mTOR signaling pathway mediated apoptosis and cell cycle arrest.

## 1. Introduction

Hepatocellular carcinoma (HCC) is the second most common cause of cancer-related death and is currently the sixth most diagnosed cancer worldwide. The global incidence varies with geographic location, but is generally higher in East Asia and Africa [1,2]. To date, chemotherapy is still the most frequently used and effective way to cure HCC. Currently, numerous first-line treatment drugs, such as sorafenib, donafenib, anlotinib, and lenvatinib have been utilized for the clinical treatment of HCC [3]. Although side effects of targeted chemotherapeutic agents are rare, there have been several cases of acquired skin perforation [4]. More seriously, HCC patients usually develop tolerance to chemotherapy drugs due to the intrinsic resistance or the acquired resistance [5]. Hence, developing notable anti-HCC ingredients derived from natural products is of great significance, and among which, plant flavonoids have been continuously attractive to researchers globally so far [6]. Studies have also shown that flavonoids exhibit good tolerance and efficacy against a variety of tumors, including liver cancer [7].

Nowadays, the regulation of apoptosis and cell cycle has attracted much attention for the treatment of cancers, such as HCC. Apoptosis, also called programmed cell death, plays a prominent role in a diversity of physiological and pathological processes [8]. Existing studies have found that apoptosis pathways include mitochondrial pathways, endoplasmic reticulum pathways, and death receptor pathways [9]. Mitochondrial apoptosis pathways are the most common, where the abnormal expression of Bcl-2 family proteins, cytochrome c, and caspases may occur [10]. Moreover, apoptosis is recognized as one of the valid strategies for tumor suppression, which involves a variety of morphological changes and cell signal transduction pathways [11]. The cell cycle is closely related to DNA replication and cell proliferation, including four phases of G0/G1, S, G2, and M. Cell cycle activities are usually regulated by cyclins (CCNs), cycle-dependent kinases (CDKs), and cyclin-dependent kinase inhibitors (CKIs) to maintain the activities of normal cell [12]. Abnormal expressions of these cell cycle factors can lead to uncontrolled cell proliferation and promote the occurrence of carcinogenesis [13]. It is well documented that more than 90% of human cancers are related to the accelerated G1 phase due to the abnormal expression of CCNs, CDKs, and CKIs [1,14]. Increasing evidence suggests that abnormal activation of the phosphatidylinositol-3-kinase (PI3K), AKT, and mammalian target of rapamycin (mTOR) pathway is a frequent event in numerous malignant tumors, including prostate cancer [15], gastrointestinal cancer [16], breast cancer [17], non-small cell lung cancer [18], acute myeloid leukemia [19], and liver cancer [20]. Recently, the activation of the PI3K/AKT/mTOR signaling pathway was found to be closely related to the regulation of apoptosis and cell cycle arrest in human endometrial cancer cells [21] and HCC [22]. A new report also showed that the natural flavonoid pectolinarigenin could induce cell apoptosis and G2/M phase cell cycle arrest of HCC by regulating the PI3K/AKT/mTOR pathway [23].

*Angelica keiskei* Koidzumi (*A. keiskei*), a traditionally healthy vegetable which is originally planted in pacific-coast islands in Japan, has been reported to contain varieties of bioactive compounds, especially chalcones [24]. It was officially recognized by the National Health Commission of China as a source of new food ingredients in 2019 after its introduction in the early 1990s, and its in-depth development and industrialization have been rapidly heating domestically ever since [25,26,27,28]. 4-hydroxyderricin (4-HD), as one of the most abundant chalcone in *A. keiskei*, exists in all parts of the plant. It has been proved to exhibit antibacterial [29], anti-inflammatory [24], antidiabetic [30,31], hypotensive [32], lipid regulation [32,33,34], and prevention of muscle atrophy [35] and loss [36], making it a valuable food-source active compound, which shows the promising potentiality of application in formulation or preparation for nutraceutical and functional foods. Specifically, in the research field of anti-cancer effects, it is reported to show anti-osteosarcoma effect by inhibiting the activation and differentiation of M2 macrophages [37]. Moreover, another study demonstrates that 4-HD can suppress melanomatogenesis by targeting BRAF and PI3K [38]. Currently, the literature relating the inhibitory effect and mechanisms of 4-HD on liver cancer is limited. Furthermore, no studies have reported the regulating roles of 4-HD on apoptosis and cell cycle arrest in HCC. Herein, we aimed to study the inhibitory effect of 4-HD on HCC and clarify the potential mechanisms by exploring PI3K/AKT/mTOR signaling pathway mediated apoptosis and cell cycle arrest.

## 2. Materials and Methods

### 2.1. Materials and Cell Culture

HepG2 and Huh7 of human HCC cell lines were supplied by the Cell Bank of the Chinese Academy of Sciences (Shanghai, China) [39]. The DMEM medium (Invitrogen, Carlsbad, CA, USA) supplemented with 10% FBS (Invitrogen, Carlsbad, CA, USA) and 1% penicillin/streptomycin (Invitrogen, Carlsbad, CA, USA) were employed for cell culture at 37 °C in a humidified incubator (5% CO_2_).

### 2.2. Cell Counting Kit-8 (CCK-8) Assay

A single-cell suspension was firstly prepared based on digestion of the HepG2 and Huh7 cells in the logarithmic growth phase with trypsin. It was then counted, seeded into a 96-well plate at a density of 5 × 10^3^ cells/mL, and placed in an incubator for culture. After the cells adhered to the wall, they were treated with 4-HD or 4-HD+LY294002 for 48 h, protected from light, and 10 µL of CCK-8 (Solarbio, Beijing, China) solution was added to each well without air bubbles. Then the cells were incubated for 30 min. Lastly, a microplate reader (Thermo Fisher Scientific, Waltham, MA, USA) was employed to read the absorbance, which was detected at 450 nm [40].

### 2.3. Wound Healing Assay

HepG2 and Huh7 cells were collected in the logarithmic growth phase and spread evenly in a six-well plate. When the cell growth density reached 95%, a 10 µL pipette tip was used to streak the line gently with a straight edge. After slowly washing with PBS, 1% serum-containing medium was added to the 6-well plate. Subsequently, 4-HD or a mixture of 4-HD and LY294002 was added for 48 h. An inverted phase-contrast microscope (Olympus, Tokyo, Japan) was used to observe cell migration.

### 2.4. Transwell Assay

The serum-free medium and Matrigel were diluted at a ratio of 1:8, added vertically to the upper chamber of the Transwell chamber (Corning, NY, USA), and placed in the incubator overnight. 2 × 10^5^ cells/mL were added to the upper chamber of the small chamber, and 4-HD, LY294002 and DMEM medium containing 20% bovine fetal serum were added to the lower chamber of the small chamber. 4% paraformaldehyde was poured into the 24-well plate, then the lower chamber was placed on a 24-well plate. Crystal violet was added to the 24-well plate after the cells were fixed for 30 min. After staining, the 24-well plate was placed under a microscope (Olympus, Tokyo, Japan) for observation.

### 2.5. Cell-Cycle Analysis

The cells were firstly cultured for 48 h. Afterwards, the cell cycle was analyzed via the cell cycle analysis kit (Beyotime, Shanghai, China). Briefly, the collected cells were fixed with cold ethanol (70%) and kept at 4 °C overnight. DNA was stained with propidium iodide (PI, 0.05 mg/mL) and RNase (2.0 mg/mL). Then, the pretreated cells were placed on the FACScan flow cytometer (BD Biosciences, San Jose, CA, USA) for cell cycle analysis. The cell percentages in G0/G1, S and G2/M phases were calculated by the Cell Lab Quanta SC software (BD Biosciences, San Jose, CA, USA).

### 2.6. Apoptosis Analysis

An Annexin V-FITC apoptosis detection kit (Sigma-Aldrich, USA) was utilized for the evaluation of the effect of 4-HD treatment on cell apoptosis via flow cytometry. HepG2 and Huh7 cells were prepared into a single cell suspension. Then it was seeded in a 6-well plate (2 × 10^5^ cells/mL) and treated with 4-HD or LY294002 for 48 h. Subsequently, the cells were digested, centrifuged, mixed with Annexin V-FITC, and then the solution of PI staining was added. Then the cells were incubated for 15 min and kept from light at room temperature. Finally, the cell apoptosis was detected by flow cytometry.

### 2.7. TUNEL Assay

TUNEL assays were implemented according to the kit manufacturer’s protocol (Abcam, USA). The HCC cells were incubated in a 48-well plate for 48 h, and then fixed by 4% paraformaldehyde. After that, 0.1% TritonX-100 was added to the 48-well plate. 3% H_2_O_2_ was used to block the cells, then Tunel reaction buffer was added, and the cells were incubated in the 37 °C incubator in the dark for 60 min. A confocal fluorescence microscope (Nikon, Tokyo, Japan) was employed to observe the cell morphology.

### 2.8. Immunofluorescence Staining

Cells were seeded on clean glass slides and treated with 4-HD or 4-HD+LY294002 for 48 h. 4% paraformaldehyde was used to fix the cells and TritonX-100 was added in 0.3% PBS to permeabilize the cells at room temperature. The following primary antibodies were used: anti-p-AKT (Abcam, Cambridge, UK). PBS contains 5% BSA was used to dilute the antibodies, and after washing, Cy3-conjugated goat anti-mouse IgG (Beyotime, Shanghai, China) was utilized to incubate the cells at room temperature for 1 h. Lastly, it was determined by a fluorescence microscope (Nikon, Tokyo, Japan).

### 2.9. Western Blotting Analysis

Western blot was performed via the standard methods. HepG2 and Huh7 cells were placed in a 6-well plate with a density of 5 × 10^5^ cells and incubated at 37 °C for 24 h. The total protein of each treatment group was extracted with lysis buffer containing 1% phenylmethanesulfonyl fluoride (PMSF). After separated, the proteins were then transferred to PVDF membranes (Solarbio, Beijing, China). The following primary antibodies were used for Western blot analysis: PI3K p85 (ABclonal A4992), Bax (ABclonal A19684), Bcl-2 (ABclonal A0208), cleaved caspase-3 (WL02348), CDK1/CDC2 (WL02373), cyclin B1 (WL01760), GAPDH (ab8245), AKT (#4685S), p-AKT (#4060S), p-PI3K p85 (#4228), m-TOR (#2983), p-mTOR (#5536), caspase-3 (#9662), caspase-9 (#9508S), cytochrome c (#11940T), PARP (#9532S), cyclin D1 (#2978P), CDK4 (#12790S), and CDK6 (#13331S). The antibodies and all secondary antibodies were supplied by CST (Danvers, MA, USA) and the membranes were visualized by BM Chemiluminescence Western Blotting Kit (Sigma-Aldrich, Schnelldoff, Germany). All the experiments were repeated three times.

### 2.10. Statistical Analysis

The results are presented as the standard error of mean ± mean, and one-way analysis of variance (ANOVA) was employed to evaluate the data. Duncan’s multiple range test was performed to compare the differences among the groups via the SPSS software (version 22.0), and statistical significance was represented by *p* < 0.05 [41]. Each experiment was repeated at least three times.

## 3. Results

### 3.1. Subsection

#### 3.1.1. 4-HD Inhibited the Proliferation and Metastasis of HepG2 and Huh7

**Figure 1:**
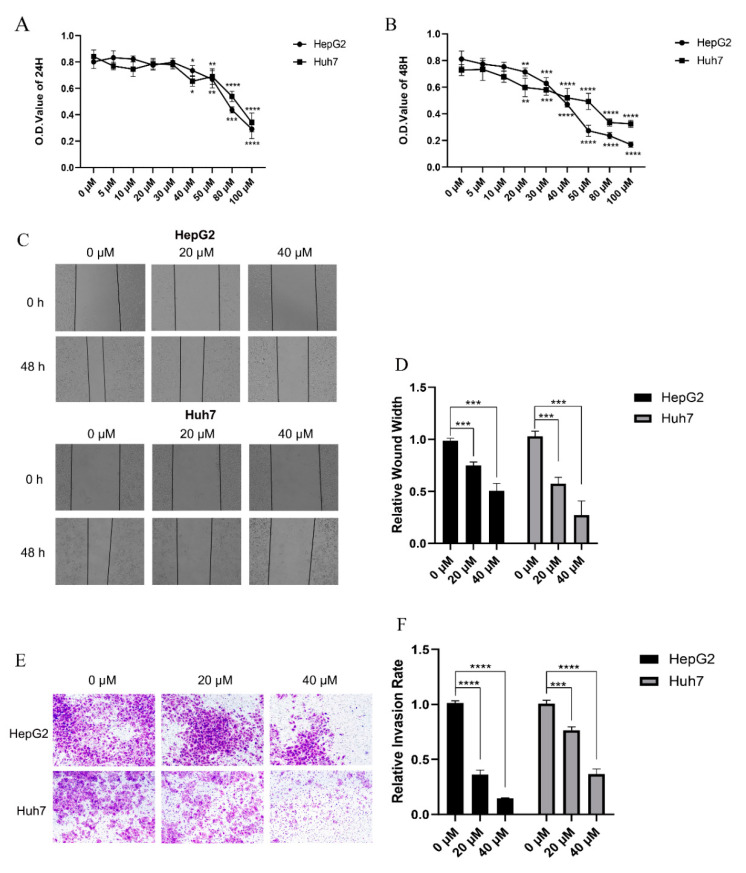
4-HD inhibits the proliferation and metastasis of HCC cells. (**A**,**B**) Determination of the survival rate of HepG2 cells and Huh7 cells treated with 4-HD (0–100 µM) after 24 h or 48 h by CCK-8 assay; (**C**,**D**) Evaluation of effects for 4-HD on cell migration by wound healing assay; (**E**,**F**) Assessment of effects for 4HD-treated cell invasion by Transwell assay. * *p* < 0.05, ** *p* < 0.01, *** *p* < 0.001, **** *p* < 0.0001.

The CCK-8 assay was employed to explore the effects of 4-HD on the cell viability of human HCC cells HepG2 and Huh7. As shown in Figure 1A,B, different concentrations of 4-HD (0, 5, 10, 20, 30, 40, 50, 80, and 100 µM) were selected and incubated with HepG2 and Huh7 cells for 24 h and 48 h, respectively. When the concentration of 4-HD was above 40 μM, the cell viability of the two types of HCC cells was significantly decreased after 24 h incubation (*p* < 0.05 for all) (Figure 1A). When the incubation was prolonged to 48 h, the cell viability was remarkably decreased by 4-HD with a concentration higher than 20 μM (*p* < 0.01 for all) (Figure 1B). A notable dose-dependent manner was observed.

The wound-healing assay was implemented to observe the migration effect of 4-HD on HCC cells. As shown in Figure 1C,D, the wound healing of HepG2 and Huh7 cells was markedly reduced after co-incubation with 20 µM and 40 µM of 4-HD for 48 h (*p* < 0.001 for all), compared with the control group, suggesting that 4-HD can effectively inhibit the migration process of HepG2 and Huh7. Subsequently, the Transwell experiment was employed to record the number of HepG2 and Huh7 cells passing through the Transwell chamber after 48 h of exposure to different concentrations of 4-HD. As seen in Figure 1E,F, the number of lower chambers was decreased when the concentration of 4-HD was raised (*p* < 0.001 for all). And obviously, 4-HD inhibited the invasion ability of HepG2 and Huh7 in a dose-dependent manner. Combining the results of wound healing and Transwell experiments, it can be inferred that 4-HD can significantly restrain the migration and invasion of HepG2 and Huh7.

#### 3.1.2. 4-HD Induced Apoptosis and Cell Cycle Arrest in HepG2 and Huh7 Cells

##### 4-HD Induced Apoptosis in HepG2 and Huh7 Cells

**Figure 2:**
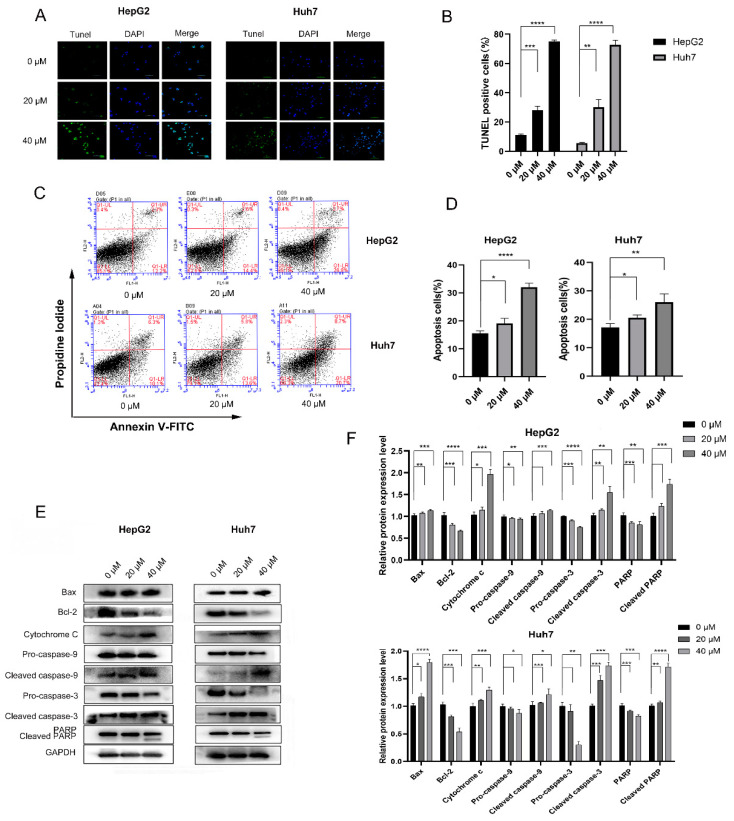
Effects of 4-HD on apoptosis of HepG2 and Huh7 cells. (**A**,**B**) TUNEL (green) and DAPI (blue) double-positive cells were elevated after treatment with various concentrations of 4-HD (magnification, 400×); (**C**,**D**) Cells were treated with 4-HD for 48 h, stained with annexin V-FITC/PI, and then analyzed by flow cytometry; (**E**,**F**) The effects of 4-HD on the expressions of Bax, Bcl-2, cytochrome c, caspase-9, caspase-3 and PARP proteins were evaluated via Western blot. Relative expressions of the proteins were normalized to GAPDH. * *p* < 0.05, ** *p* < 0.01, *** *p* < 0.001, **** *p* < 0.0001.

To further evaluate the pro-apoptosis effects of 4-HD on HepG2 and Huh7 cells, TUNEL and flow cytometry were successively performed. As shown in Figure 2A,B, the number of positive cells in the 4-HD treatment group was increased in a dose-dependent manner (*p* < 0.01 for all), compared with the control group. It can be observed from Figure 2C,D that, as the concentrations of 4-HD went up, the rate of apoptosis exhibited an upward trend (*p* < 0.05 for all). Furthermore, Western blot experiments were employed to explore the mechanism of 4-HD-induced apoptosis of HepG2 and Huh7 cells. As seen in Figure 2E,F, the expressions of cytochrome c, cleaved caspase-3, cleaved caspase-9, cleaved PARP, and Bax proteins were up-regulated as the concentrations of 4-HD rose, while the expressions of pro-caspase-3, pro-caspase-9, PARP, and Bcl-2 proteins were remarkably down-regulated (*p* < 0.05 for all). These results indicate that 4-HD can promote HepG2 and Huh7 cells apoptosis by activating the mitochondrial apoptosis pathway.

##### 4-HD Induced Cell Cycle Arrest in HCC Cells

**Figure 3:**
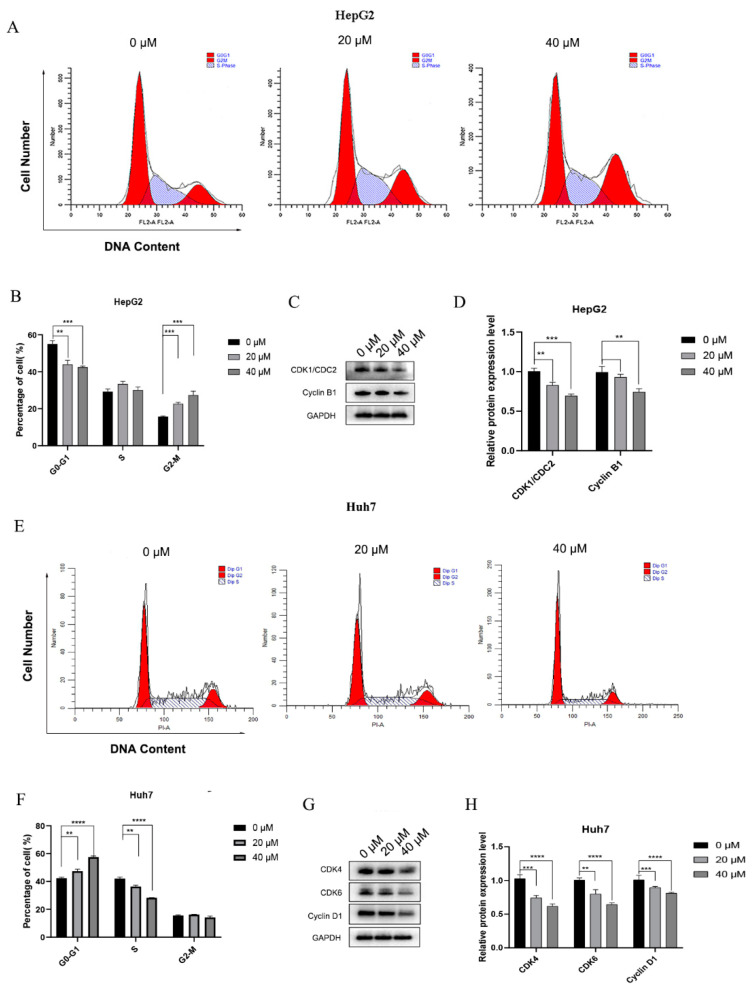
The effect of 4-HD on HCC cell cycle distribution. (**A**,**B**) Phase distribution of HepG2 treated with 4-HD for 48 h by flow cytometry analysis; (**C**,**D**) The effects of 4-HD on the expression of Cyclin B1 and CDK1/CDC2 in HepG2 cells were evaluated by Western blot; (**E**,**F**) Phase distribution of Huh7 treated with 4-HD for 48 h by flow cytometry analysis; (**G**,**H**) The effects of 4-HD on the expressions of Cyclin D1, CDK4 and CDK6 in Huh7 cells were evaluated by Western blot. Relative expressions of the proteins were normalized to GAPDH. ** *p* < 0.01, *** *p* < 0.001, **** *p* < 0.0001.

Cells can be divided into the G0/G1, S, and G2/M phases according to the DNA content and detected by flow cytometry. Herein, the effects of 4-HD on the cell cycle of HepG2 and Huh7 cells were investigated. After PI staining, the cells in different cell cycles were distinguished according to the fluorescence intensity. As shown in Figure 3A,B,E,F, the proportion of HepG2 cells in the G2/M phase was increased with the elevation of 4-HD concentrations. However, the proportion of Huh7 cells in the G0/G1 phase was surprisingly increased as well, suggesting that 4-HD can block HepG2 cells in the G2/M phase and Huh7 cells in the G0/G1 phase (*p* < 0.01 for all).

HepG2 and Huh7 cells were respectively incubated with 4-HD at concentrations of 20 µM and 40 µM for 48 h, and the proteins were extracted. As displayed in Figure 3C,D,G,H, the relative protein expressions of cyclin B1 and CDK1/CDC2 were determined. In HepG2 cells, the levels of cyclin B1 and CDK1/CDC2 were declined with the increasing concentrations of 4-HD, while in Huh7 cells, the expressions of cyclin D1, CDK4, and CDK6 were dramatically down-regulated. It can be inferred that 4-HD can induce HepG2 cells arrest at the G2/M phase and Huh7 cells arrest at the G0/G1 phase (*p* < 0.01 for all).

#### 3.1.3. 4-HD Regulated the PI3K/AKT/mTOR Pathway

**Figure 4:**
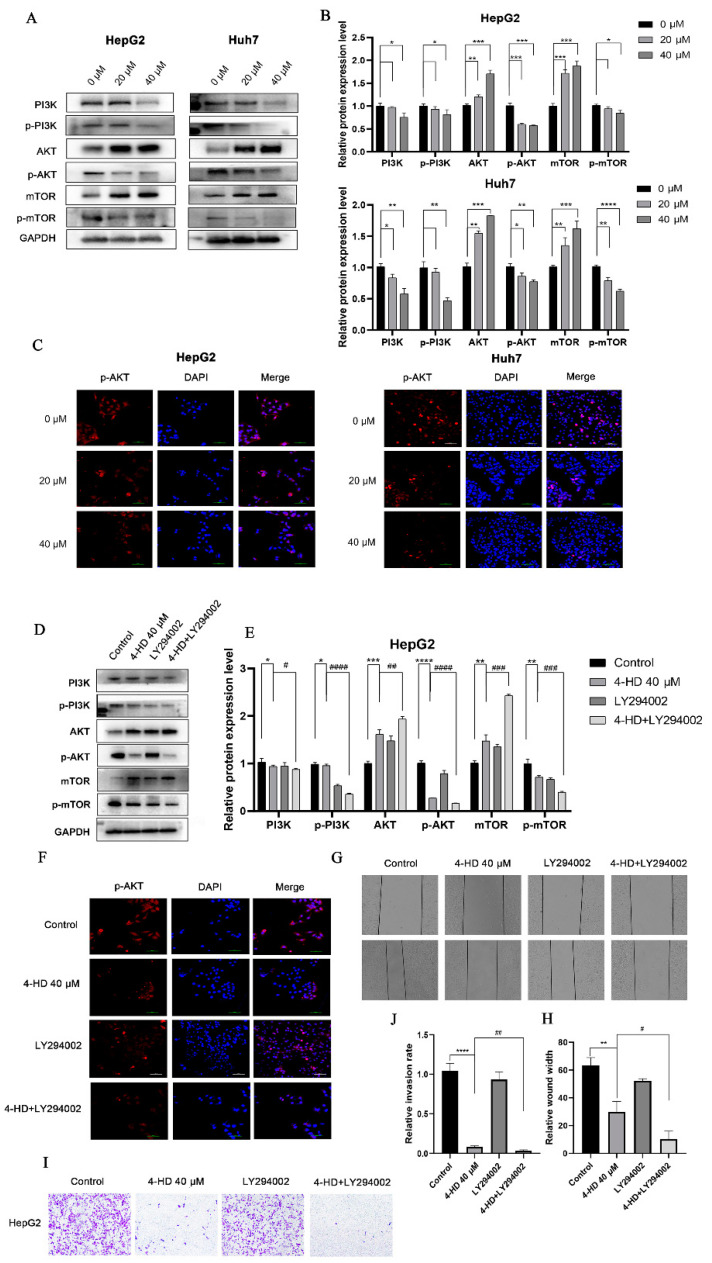
4-HD inhibited the proliferation and metastasis of HCC cells by regulating the PI3K/AKT/mTOR signaling pathway. (**A**,**B**) Relative proteins expressions of PI3K/AKT/mTOR pathway in HepG2 cells and Huh7 cells treated with 0 μM, 20 μM, and 40 μM of 4-HD for 48 h; (**C**) Immunofluorescence was employed to quantify the expression of p-AKT protein in HepG2 cells (magnification: 400×); (**D**,**E**) Relative proteins expressions of PI3K/AKT/mTOR pathway in HepG2 cells were treated with PBS (control), 4-HD (40 μM 4-HD), LY294002 (10 μM LY294002) and 4-HD + LY294002 (40 μM 4-HD + 10 μM LY294002) for 48 h; (**F**) immunofluorescence was performed to quantify the expression of p-AKT protein in HepG2 cells treated with LY294002 (magnification: 400×); (**G**,**H**) wound healing assay was carried out to conduct the effect of LY294002 on the migration of HepG2 cells; (**I**,**J**) Transwell assay was implemented to detect the effect of LY294002 on the invasion of HepG2 cells. * *p* < 0.05, ** *p* < 0.01, *** *p* < 0.001, **** *p* < 0.0001 vs. control (0 μM); ^#^ *p* < 0.05, ^##^ *p* < 0.01, ^###^ *p* < 0.001, ^####^ *p* < 0.0001 vs. 40 μM 4-HD.

To investigate that 4-HD regulates the PI3K/AKT/mTOR pathway, the protein expressions of genes related to the pathway were detected. It can be observed from Figure 4A,B that, as the concentration of 4-HD raised, the expressions of PI3K, p-PI3K, p-AKT, and p-mTOR proteins were down-regulated, while the expressions of AKT and mTOR proteins were up-regulated (*p* < 0.05 for all). Moreover, the same results were obtained in the immunofluorescence experiment, as shown in Figure 4C, indicating that 4-HD can inhibit the phosphorylation of AKT.

In order to confirm whether 4-HD governed the PI3K/AKT/mTOR axis, PI3K inhibitor LY294002 was selected, Western blot analysis and immunofluorescence experiments were performed to detect related proteins. Figure 4D,E shows that the expressions of p-PI3K, p-AKT, and p-mTOR proteins in the 4-HD+LY294002 group were notably decreased compared with the 4-HD group (*p* < 0.05 for all). As seen in Figure 4F from immunofluorescence results, the expressions of p-AKT gradually were decreased, compared with the control group. What’s more, the inhibitory effect of p-AKT was enhanced by LY294002+4HD group.

Then, aiming to validate whether the PI3K/AKT/mTOR signaling pathway was involved in 4-HD inhibiting the migration and invasion of HCC cells, LY294002 was incubated with HepG2 cells, followed by wound healing and Transwell experiments. It can be observed from Figure 4G,H that the wound healing width of 4-HD and LY294002 combined treatment group was markedly higher than that of LY294002 alone treatment group, indicating that LY294002 combined treatment can enhance the inhibition of cell migration (*p* < 0.05 for all). As noticed in Figure 4I,J, the number of HepG2 cells that invaded the lower chamber in the 4-HD and LY294002 combination group was eminently lower than that in the 4-HD group (*p* < 0.01 for all). It can be inferred from these results that 4-HD can inhibit the proliferation and metastasis of HCC cells by regulating the PI3K/AKT/mTOR pathway.

#### 3.1.4. 4-HD Induced Apoptosis and Cycle Arrest of HCC Cells by Regulating the PI3K/AKT/mTOR Pathway

**Figure 5:**
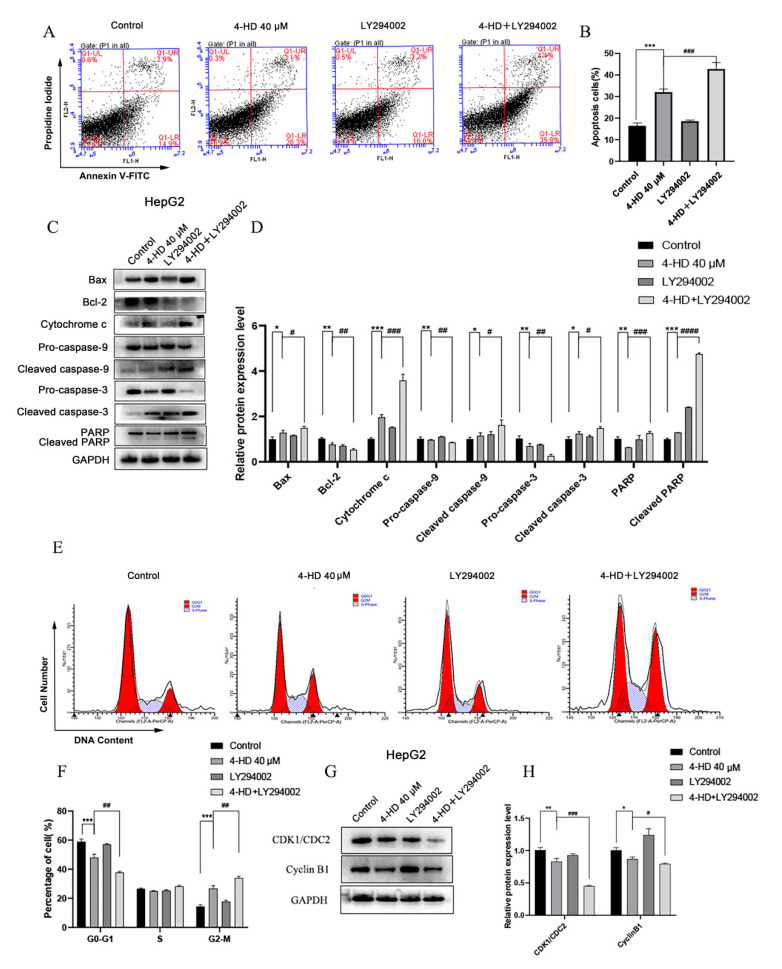
4-HD induced apoptosis and cycle arrest of HCC cells by regulating the PI3K/AKT/mTOR signaling pathway. (**A**,**B**) Cell apoptosis proportion treated with 4-HD+LY294002 was detected by flow cytometry; (**C**,**D**) The effects of LY294002 on the expressions of Bax, Bcl-2, cytochrome c, caspase-9 and caspase-3 and PARP in HepG2 cells treated with 4-HD were evaluated by Western blotting; (**E**,**F**) Cell cycle distribution proportion treated with 4-HD+LY294002 was determined by flow cytometry; (**G**,**H**) Effects of LY294002 on the expressions of cyclin B1 and CDK1/CDC2 proteins in HepG2 cells treated with 4-HD were assessed by Western blotting. Relative expressions of the proteins were normalized to GAPDH. * *p* < 0.05, ** *p* < 0.01, *** *p* < 0.001 vs. control (0 μM); ^#^ *p* < 0.05, ^##^ *p* < 0.01, ^###^ *p* < 0.001, ^####^ *p* < 0.0001 vs. 40 μM 4-HD.

To further verify whether PI3K/Akt/mTOR signaling pathway is involved in the process of 4-HD inducing apoptosis and cell cycle arrest of hepatoma cells, as shown in Figure 5A,B, the proportion of apoptosis in the 4-HD + LY294002 treatment group was increased, compared with the control group (*p* < 0.001). As seen in Figure 5C,D, compared with the 4-HD group, the expressions of cytochrome c, cleaved caspase-3, cleaved caspase-9, cleaved PARP, and Bax proteins were remarkably elevated in the 4-HD + LY294002 group, accompanied by the decreasing expressions of pro-caspase-3, pro-caspase-9, PARP and Bcl-2 proteins (*p* < 0.05 for all).

The cell cycle distribution of HepG2 was assessed by flow cytometry. As exhibited in Figure 5E,F, compared with the 4-HD group, the cell proportion at the G0/G1 phase was declined, while the one at the G2/M phase was increased after combining with LY294002 (*p* < 0.01 for all). Furthermore, Western blot was performed to verify the expression of cyclin. As shown in Figure 5G,H, 4-HD+LY294002 treatment significantly down-regulated the expressions of CDK1/CDC2 and Cyclin B1 proteins (*p* < 0.05 for all). Combining the above experimental data, it can be concluded that 4-HD can induce apoptosis and cycle arrest of HCC cells through the PI3K/AKT/mTOR signaling axis.

## 4. Discussion

4-HD is one of the major natural chalcone isolated from *A. keiskei* with various functional properties, such as anti-tumor. To our knowledge, this is the first study evaluating the inhibitory effects of 4-HD on HCC cells. In two typical HCC cell lines, HepG2 and Huh7, we found 4-HD induced remarkable cell cycle arrest and apoptosis along with the inhibitory effect on the proliferation and metastasis. What’s more, we proved that 4-HD may promote apoptosis and cell cycle arrest of the HCC cells by modulating the PI3K/AKT/mTOR signaling pathway.

Apoptosis is the main way of programmed cell death, which can restrain tumor cell growth [42]. Drug-induced cell apoptosis is mainly regulated by the mitochondrial mechanism through caspase activations [43]. Caspase and Bcl-2 family genes play an important regulatory role in the process of cell apoptosis [44]. Caspase-3 regulates the entire process of cell apoptosis [45]. Bcl-2 is a membrane protein that inhibits the release of cytochrome c (Cyto-C) by regulating the permeability of the mitochondrial membrane and restrains the activation of caspase-3 to exert anti-apoptotic effects [46]. Bax, as a pro-apoptotic protein, can be suppressed by forming a dimer with Bcl-2, while its activation destroys the integrity of the mitochondrial membrane. Subsequently, cyto-C is released from the mitochondrial membrane, which activates caspase-3 and induces mitochondrial apoptosis [47]. Our results showed that the expressions of pro-apoptotic proteins were up-regulated while those of anti-apoptotic proteins were down-regulated by 4-HD treatment. A previous study has shown that 4-HD can induce apoptotic death of HL60 cells through death receptor-mediated and mitochondrial pathways [48], which is consistent with our findings that 4-HD induces mitochondrial apoptotic cell death to exert anti-HCC cell proliferation effects.

The cell cycle is regulated by a protein complex composed of cyclins and cyclin-dependent kinases (CDK). It has been reported that the abnormal levels of CDK4, CDK6, and cyclin D1 in various human cancer cells are closely related to the abnormal proliferation of cancer cells [49,50]. Cell cycle arrest is considered as a potential target for cancer therapy in numerous malignant cancers [51]. Decrease in cyclin D inhibits growth and induces cell death in tumors such as esophageal, colon, and pancreatic cancers [52,53,54]. Many chemotherapeutic drugs exhibit anti-tumor effects by inducing cell cycle arrest [55]. The activation of CDK1/CDC2 and cyclin B1 plays a key role in the initial stages of mitosis. And the existing document indicates that the down-regulation of CDK1/CDC2 and cyclin B1 is related to the G2/M cycle arrest [56]. In this study, the expressions of cyclin B1 and CDK1/CDC2 proteins in HepG2 cells treated with 4-HD were down-regulated, while those of cyclin D1 and CDK4 were down-regulated in Huh7 cells, suggesting that 4-HD can trigger HepG2 cells arrest at G2/M phase and Huh7 cells arrest at G0/G1 phase.

**Figure 6:**
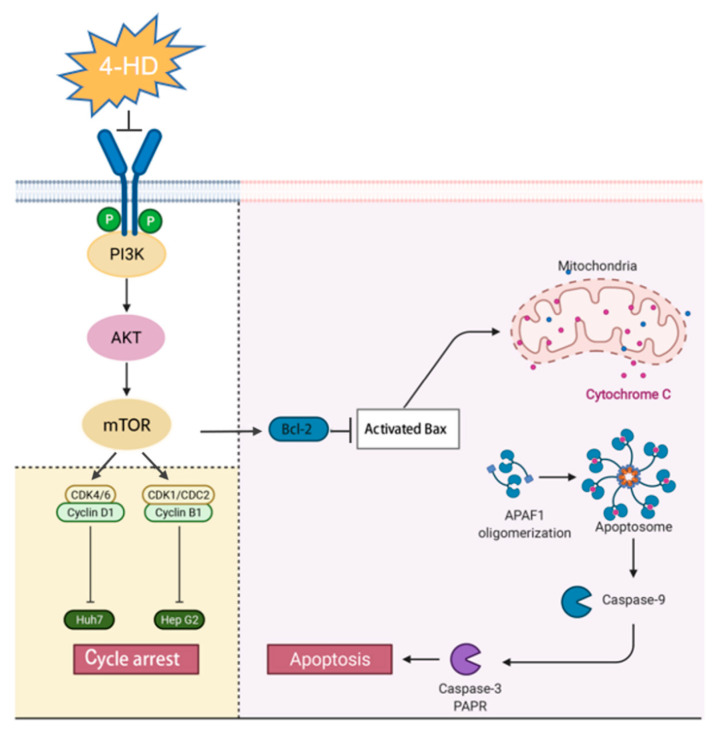
Proposed mechanism for 4-HD inducing apoptosis and cycle arrest in HCC cells through the PI3K/AKT/m-TOR signaling pathway. ⊥ indicates an inhibitory effect and → indicates a promoting effect.

PI3K/AKT/m-TOR cascade is a signal transduction pathway that regulates cancer cell growth, proliferation, cell energy, proliferation, senescence, and angiogenesis [57]. Inhibiting the different processes of the PI3K/AKT/m-TOR pathway is a common strategy for the treatment of human malignant tumors [58]. Many bioactive flavonoids, such as collagen and paclitaxel, have been reported to down-regulate the expressions of proteins such as p-PI3K, p-AKT, and p-mTOR by inhibiting PI3K/AKT/mTOR pathway in HCC [59,60]. Our study shows that the protein levels of PI3K, p-PI3K, p-AKT, and p-mTOR were down-regulated following 4-HD treatment, while the expressions of AKT and mTOR proteins were up-regulated, indicating that the suppression effect of 4-HD on the PI3K/AKT/mTOR signaling pathway. Moreover, we also co-treated the HCC cells with both 4-HD and LY294002, an inhibitor of PI3K. Both apoptosis and cycle arrest were exacerbated in the co-treatment groups. A previous study demonstrated that 4-HD could promote apoptosis and induces cycle arrest in melanoma by targeting PI3K [32], which complies with our findings. Collectively, all these results indicated that 4-HD induced apoptosis and cell cycle arrest of the HCC cells by modulating the PI3K/AKT/mTOR signaling pathway to inhibit the proliferation of HCC cells (Figure 6).

## 5. Conclusions

In summary, this study revealed that 4-HD exhibited proliferation inhibitory effects in HepG2 and Huh7 cells in a dose-dependent manner. The potential mechanism may be related to the inhibition of the PI3K/AKT/m-TOR signaling pathway and the subsequent inducing of mitochondrial apoptosis and cell cycle arrest. This study provides a new strategy for the therapy of HCC and a theoretical basis for the exploiting of 4-HD as an anti-hepatoma natural functional ingredient.

## Data Availability

All of the data are presented in the manuscript.
